# Strategies to Target Specific Components of the Ubiquitin Conjugation/Deconjugation Machinery

**DOI:** 10.3389/fchem.2019.00914

**Published:** 2020-01-10

**Authors:** Neil C. Taylor, Joanna F. McGouran

**Affiliations:** School of Chemistry and Trinity Biomedical Sciences Institute, Trinity College Dublin, Dublin, Ireland

**Keywords:** activity-based protein profiling, ubiquitin, protein modification, deubiquitinating enzymes, probe

## Abstract

The regulation of ubiquitination status in the cell is controlled by ubiquitin ligases acting in tandem with deubiquitinating enzymes. Ubiquitination controls many key processes in the cell from division to death making its tight regulation key to optimal cell function. Activity based protein profiling has emerged as a powerful technique to study these important enzymes. With around 100 deubiquitinating enzymes and 600 ubiquitin ligases in the human genome targeting a subclass of these enzymes or even a single enzyme is a compelling strategy to unpick this complex system. In this review we will discuss different approaches adopted, including activity-based probes centered around ubiquitin-protein, ubiquitin-peptide and mutated ubiquitin scaffolds. We examine challenges faced and opportunities presented to increase specificity in activity-based protein profiling of the ubiquitin conjugation/deconjugation machinery.

## Introduction

Ubiquitin is a small protein that is added post-translationally to substrate proteins, modulating their activity and interactions (Goldstein et al., 1975). It has a major role in DNA repair (Jentsch et al., 1987), transcriptional regulation (Hochstrasser and Varshavsky, 1990), cell cycle (Ciechanover et al., 1984; Finley et al., 1984), and stress responses (Ciechanover et al., 1984; Finley et al., 1984) amongst others. Ubiquitin is added to substrate proteins *via* E1, E2, and E3 enzymes (Ciechanover et al., 1982; Hershko et al., 1983) activating, conjugating and ligating ubiquitin, culminating in isopeptide bond formation between a lysine residue of the substrate protein and the C-terminus of ubiquitin (Hunt and Dayhoff, 1977).

Substrates can be modified with monoubiquitin (Haglund et al., 2003; Carter et al., 2007) or polyubiquitin chains linked by isopeptide bonds between an ubiquitin C-terminus and one of the seven lysine residues or N-terminus of another ubiquitin (Hershko and Heller, 1985). The linkage types afford distinct topologies, essential in determining the substrate protein's fate (Chau et al., 1989; Peng et al., 2003; Xu et al., 2009).

Deubiquitinating enzymes (DUBs) possess ubiquitin C-terminal hydrolytic activity, removing ubiquitin (Pickart and Rose, 1985; Hough and Rechsteiner, 1986). The human genome encodes ~100 DUBs, split into six families; ubiquitin-specific proteases (USPs), ubiquitin C-terminal hydrolases (UCHs), ovarian tumor proteases (OTUs), Machado-Josephin domain proteases (MJDs), the JAB1/MPN/MOV34 family (JAMMs) and the motif interacting with Ub-containing novel DUB (MINDY) family. All families, excluding the JAMM zinc metalloproteases, are cysteine proteases and will be the focus of this review (Hanpude et al., 2015; Abdul Rehman et al., 2016). Given the diversity in ubiquitin chain length, linkage type and protein substrate, DUB specificity is key to biological function.

Dysregulation of the enzymes involved in ubiquitin signaling can result in disease states. Genes encoding the DUBs CYLD and BAP1 are established tumor suppressor genes, often mutated in cancer phenotypes (Zhao et al., 2011). Additionally, members of the OTU family are upregulated in several cancer types (Carneiro et al., 2014). There is also a growing number of studies linking DUBs to neurological diseases (Bhattacharyya et al., 2012; Imai et al., 2012; Xilouri et al., 2012). Mutations in members of the ubiquitin cascade have been implicated in similar disorders (Bernassola et al., 2008; Popovic et al., 2014). The importance of these enzymes in cellular processes and disease states has created demand for molecular tools to assist their study.

Activity-based probes target only the active form of an enzyme allowing for the identification and characterization of active enzymes within complex cellular milieus. They provide a more accurate picture of an enzyme's influence in a cell in comparison to traditional transcriptomic or proteomic screens which do not account for differences in activity, caused by post translational modifications or other inhibitory effects. Probes targeting DUBs based on monoubiquitin have been successful in characterizing new DUB family members (Borodovsky et al., 2002) aiding the crystallization of DUBs (Misaghi et al., 2005) and assessing novel DUB inhibitors (Kramer et al., 2012). The first example of an activity-based probe targeting DUBs consisted of a vinyl sulfone “warhead” in place of the C-terminal glycine residue of ubiquitin (Borodovsky et al., 2001). A variety of thiol-reactive electrophiles have since been reported (Borodovsky et al., 2002; Love et al., 2009; Ekkebus et al., 2013; de Jong et al., 2017). These probes provide information about global DUB activity with some also shown to react with members of the conjugation machinery (Mulder et al., 2016). Recently, large biological screens using these probes have aided in the development of a new chemoproteomic method that could potentially be used to identify the labeling site of any covalent modifier (Hewings et al., 2018). A similar screen demonstrated how chemoproteomics can be used to study DUBs in a more comprehensive manner (Pinto-Fernández et al., 2019). These examples elegantly demonstrate the depth of knowledge that can be obtained using these probes.

Recently, focus has shifted toward the development of probes to target specific subsets of ubiquitin conjugation/deconjugation machinery to allow for more precise investigations of their activity. Using the knowledge that the binding domain recognizing ubiquitin, the C-terminal adduct, chain length and linkage type all affect the specificity of these enzymes, new generations of probe have been developed. The generation of selective probes harbors significant challenges and this review will focus on the design and synthesis of probes to tackle this problem.

## Probes for Deubiquitinating Enzymes

### Mutated Ubiquitin Probes

The binding interactions of DUBs are mediated by ubiquitin-binding domains. An innovative strategy based on mutation of WT ubiquitin to enhance/diminish specific interactions between ubiquitin and DUB binding domains was developed by Ernst et al. (2013). It involved random mutation of Ubiquitin and selection through phage display assays. Although the ubiquitin variants were able to pull out endogenous deubiquitinating enzymes in a selective manner their primary design and use was for inhibition or enhancement of endogenous DUB/Ligase activity though cellular expression. Several DUBS and ligases have been targeted in this manner (Zhang et al., 2013, 2016; Gabrielsen et al., 2017; Gorelik and Sidhu, 2017). Ovaa and co-workers extended this methodology to generate USP7 selective activity-based probes using ubiquitin variants developed by Zhang et al. (Ernst et al., 2013; Zhang et al., 2013, 2016) and computational models as starting points for mutations (Gjonaj et al., 2019). Probes incorporated a C-terminal alkyne warhead for covalent capture and an N-terminal Rhodamine dye. Rounds of screening were monitored by probe labeling of HAP1 cell lysate. Interestingly low reactivity was seen for mutants reported to be strong binders in previous phage display assays (Zhang et al., 2013, 2016; Gabrielsen et al., 2017), potentially due to incorrect alignment of the warhead in these variants (Gjonaj et al., 2019). However, an iterative approach screening >120 total variants afforded a probe with high USP7 selectivity.

### Ubiquitin-Peptide Probes

DUBs show specificity toward different chain linkage types and the substrate protein. Therefore, extending the probe scaffold by appending an ubiquitin or target protein peptide onto the C-terminus of a Ubiquitin probe beyond the electrophilic trap can increase specificity.

The first example by Iphöfer et al. generated ubiquitin linkage mimics (Iphofer et al., 2012). Peptide sequences were coupled to a warhead containing linker followed by reaction with HA-Ub_75_-thioester. Probes with peptide sequences reflecting the K48 and K63 regions were tested in Jurkat cell lysate. Differences in labeling were seen between the probes which were both restricted in comparison to the Ub-VME probe. These probes were the first step toward determining DUB selectivity using an activity-based probe approach. The strategy is broadly applicable and laid excellent groundwork but results in a linker two atoms longer than the natural substrate and, as with all peptide probes, selectivity determined by the tertiary structure of the substrate protein is lost.

A further example of this probe type was developed by the Chatrerjee laboratory in 2016 using a selenocystine ligation (Whedon et al., 2016). The approach is similar to that developed by Brik and co-workers (Haj-Yahya et al., 2014), however the use of selenocysteine allowed for Cysteine residues within the peptide. A peptide centered on K117 of TRIM25 was used, containing two Cys residues and a Met alongside the SeCys introduced at position 117. Ligation of the selenium with Ub_75_-thioester and subsequent Se to N acyl shift resulted in the ubiquitinated peptide bearing a SeCys at Ub_76_. Selective alkylation of Selenium at low pH afforded the DHA probe in the presence of Cys residues. TRIM25 is known to be deubiquitiniated by USP15 suggesting potential USP15 probe selectivity, however this was not investigated. Reactivity was demonstrated with recombinant USP15, showing predominantly active site labeling. This work extended the DHA methodology to allow the presence of cysteine in peptides/proteins. The low pH (3.4) of the selective alkylation could however limit the utility for protein conjugation. Also, the presence of a native isopeptide bond in the scissile position may affect probe stability in complex systems.

### Diubiquitin Probes

Shortly following Iphöfer et al. several diubiquitin probes were created, further extending the probe scaffold. McGouran et al. generated the first full length diubiquitin probes (McGouran et al., 2013). A “warhead” bearing an alkyne handle was coupled to HA-Ub_75_-thioester. An azidohomoalanine incorporated into the proximal ubiquitin allowed triazole formation to generate the probes. All linkage types were mimicked and probe selectivity was quantified in HEK293T cell lysate. Distinct labeling profiles were observed between the probes without selectivity for a single DUB. The method is broadly applicable, although incompatible with multiple methionine residues. The linker is four atoms longer than in the natural substrate but is uncleavable, providing a robust probe.

**Figure 1:**
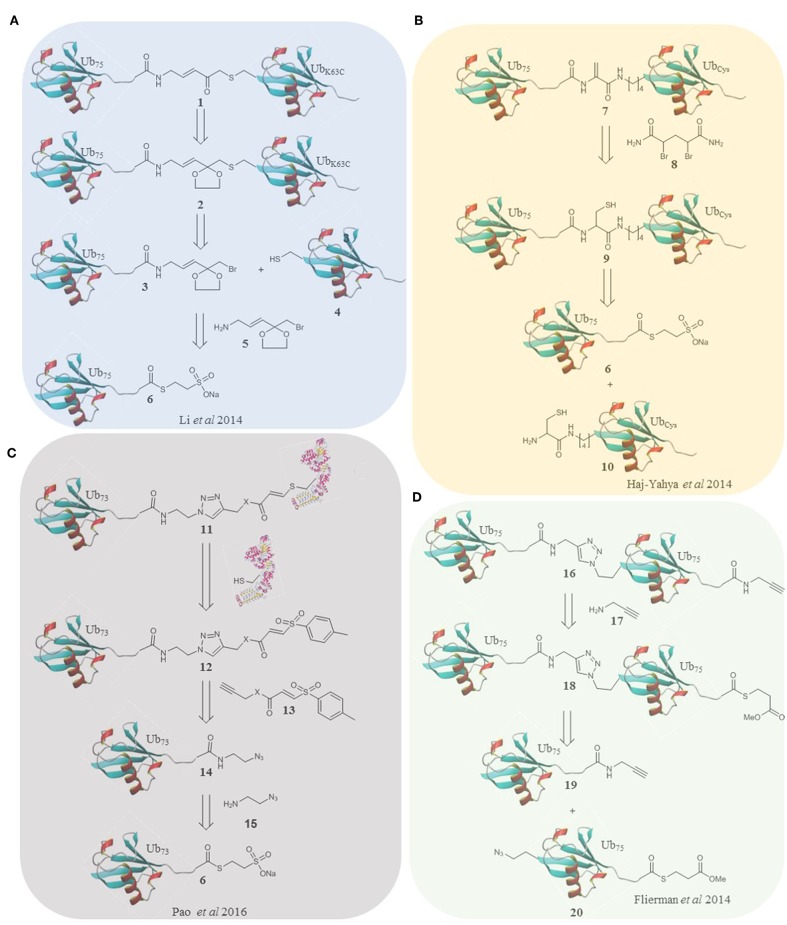
Retrosynthesis of selected probes. **(A)** A methodology reported by Li et al. utilizes an α-bromo-vinylketal to link Ub_75_ thioester **6** and the mutated Ub monomer **4**. Deprotection of the ketal of 2 unmasks a Michael acceptor within the linker of the probe **1**. **(B)** Haj-Yahya et al. synthesized a diubiquitin probe based on DHA as the electrophilic warhead. NCL is used to link Ub monomers **6** and **10**, positioning a cysteine residue at position 76 of the distal Ub which is then converted to DHA using the dibromide reagent **7**. **(C)** Pao et al. expanded on the TDAE methodology to incorporate a Ub monomer and E2 enzyme in a single probe. Alkyne functionalized TDAE **13** is coupled to azido functionalized Ub monomer **14** using copper catalyzed cycloaddition. A subsequent reaction with an E2 enzyme eliminates the tosyl component of the TDAE **12**, affording the final probe **11** containing a Michael acceptor. **(D)** Flieman et al. use copper catalyzed cycloaddition to conjugate two modified Ub monomers **19** and **20**. Propargyl amine **17** was reacted with the C-terminus of the proximal monomer to yield probe **16**.

In 2014 the Zhuang laboratory developed an alternative method using a warhead bearing a sulfur reactive group (Li et al., 2014). This was coupled to Ub_75_-thioester. The proximal HA-ubiquitin containing a single cysteine at the 48/63 position was reacted to generate two diubiquitin activity-based probes (Figure 1A). The probes were tested in HEK293T cell lysate again giving distinct labeling profiles. This elegant method affords a non-hydrolyzable linker of the correct length, although it is incompatible with multiple cysteine residues. Recently, this probe was one of a panel that were used to report the mechanism by which USP9X recognizes substrates in a linkage specific manner by using a combination of activity-based labeling and crystallization studies. This study described previously unreported mechanistic and structural recognition features of these enzymes showing how these probes provide a useful insight into enzyme activity (Paudel et al., 2019).

Brik and co-workers took a strategy using dehydroalanine formation (Haj-Yahya et al., 2014), based on their previous non-cleavable diubiquitin synthesis (Kumar et al., 2010). Native chemical ligation and desulfurization to furnish the electrophilic trap in the form of a DHA gave the linear, 48 and 63 linked probes. To accomplish this, the relevant nitrogen of the proximal ubiquitin was selectively deprotected and coupled to a protected cysteine. After deprotection the sulfur reacts with Ub_75_-thioester followed by an S to N acyl shift and dehydroalanine formation (Figure 1B). In this probe design, and all subsequent probes based on this strategy, the native isopeptide bond is still present and the electrophilic trap is two (branched probes) or three (linear probe) atoms from the native position. These probes could therefore either trap or be cleaved by active DUBs. Interestingly the K63 probes labeled recombinant DUBs and the linear probe showed only cleavage with the DUBs tested. The K48 probe showed both labeling and cleavage.

Ovaa and co-workers also utilized the elimination of sulfur to give a Michael acceptor in their final step (Mulder et al., 2014). Orthogonally protected diaminnocutyric acid replaced the lysine residue of interest. A short sulfur containing linker was coupled prior to ligation and desulfurization to afford the diubiquitin probes. This was carried out for all 7 lysine linkages and affords a linkage that matches the native length and is not degraded by DUBs. All probes were tested with recombinant DUBs, the K11 and K48 probes were also tested in EL4 lysate. Both were seen to display a restricted labeling pattern in comparison to the VME probe and were later used to characterize Cezanne (Mevissen et al., 2016). In addition to this, the probe was used to elucidate the linkage specificity of Mug105, which along with ZUSFP, was identified as a founding member of a novel family of DUBs (Hermanns et al., 2018).

Although all the Diubiquitin probes demonstrated more selective labeling patterns than mono ubiquitin probes the linear probe generated by Krappmann and co-workers (Weber et al., 2017) was the first to show single DUB selectivity. Using an approach similar to Brik and co-workers, an N-terminal cysteine was introduced to the proximal ubiquitin allowing native chemical ligation to a Ub_75_-thioester and desulfurization to afford a dehydroalanine war head. This resulted in a linear probe with the native linker length and an electrophilic trap one bond away from the scissile peptide bond. This probe structure, once optimized by removal of the C-terminal glycine, proved to be selective for OTULIN in cell lysate (Weber et al., 2017).

Li and co-workers used photoaffinity labeling for their K27 linked diubiquitin probe (Tan et al., 2017). They took a native chemical ligation approach using a biotinylated proximal ubiquitin functionalized at K27 with a cysteine coupled to the ε-N. This was ligated to Ub_75_-NH_2_NH_2_ to afford the native isopeptide bond adjacent to a single cysteine. Sulfur alkylation installed the photo crosslinking group to the probes. A slightly broader reactivity profile was seen in comparison to the corresponding DHA probe in HEK293F lysate. The synthetic method could be easily applied to other systems and resulted in a native linker length. Due to the nature of photo-crosslinking, proteins which bind K27 linked ubiquitin can also be detected and the presence of the native isopeptide bond gives the possibility of cleavage of the probe. As the photo-crosslinking doesn't require an active site cysteine this method can also profile metalloprotease DUBs.

In 2011 Ye et al. generated a noncleavable linear diubiquitin with a C-terminal aldehyde via expression of a diubiquitn-intein construct (Ye et al., 2011). This aided study of USP21 by crystallization although its potential as an activity based probe was not explored.

In 2016 Ovaa and co-workers fully expanded the diubiquitin probe concept to probe the S1-S2 pocket of DUBs (Flierman et al., 2016). To this end they generated triazole linked non-cleavable diubiquitins bearing a C-terminal thioester on the proximal ubiquitin. A propargyl warhead was introduced to generate the probe (Figure 1D). This design allowed examination of DUB activity for diubiquitin binding in the S1-S2 pocket without degradation of the probe should it enter the S1'-S1 pocket. All 7 lysine linked diubiquitins were generated and the K6, 11 & 48 probes were tested in EL4 cells, showing different labeling patterns. DUBs with low reported specificity when probing the S1'-S1 pocket can display specificity in probing the S1-S2 pocket as demonstrated by the SARS PLpro DUB (Bekes et al., 2016). This was the first time a DUB was proven to be specific for K48 linked chains over monoubiquitin, exhibiting how these probes are superior, at least in some cases, at elucidating poly ubiquitin linkage specificity in DUBs.

In 2019, Tong and co-workers extended the concept a step further by generating triubiquitin activity-based probes. The probes bear a native isopeptide bond between the proximal and middle ubiquitin and a warhead between the middle and distal ubiquitin (Paudel et al., 2019). The proximal K63 linked diubiquitin was generated enzymatically with the middle ubiquitin harboring a K63C mutation to allow ligation through the same methodology as employed by the Zhuang lab (Li et al., 2014). These probes gave insights into the binding modes of USP9X. Although using the probes in more complex milieus would be complicated by the cleavable isopeptide bond it demonstrates the limitless scope of such approaches (Paudel et al., 2019).

### Ubiquitin-Protein Conjugate Probes for Deubiquitinating Enzymes

To further unpick DUB specificity the challenging aim of generating Ubiquitin-protein conjugate probes has been addressed. In 2018 Brick (Meledin et al., 2018) and Zhuang (Gong et al., 2018) both extended their conjugation methodologies to create Ub-protein probes. Brik and co-workers targeted ubiquitinated α-globin, forming DHA at the single cysteine (104) present in α-globin and coupling to a thiol bearing thiazolidine. Deprotection allowed ligation to Biotin-Ub_75_-thioester. A further DHA formation step installed the electrophilic trap into the Ubiquitin-α-globin conjugate. This strategy utilizes the single cysteine present in α-globin and its proximity to a ubiquitination site at K100. Quantitation of probe activity in erythrocyte lysate detected enrichment of several DUBs including USP15, which was confirmed with the natural substrate and was also shown to deubiquitinate the K119 position in an independent study (Sun et al., 2018).

This methodology was extended by the development of selective deprotection of three cysteine protecting groups using palladium species in order to make a Ubiquitinated Histone probe (Jbara et al., 2018). The multiple cysteines present allowed NCL to build the target protein which was desulfurised prior to release of the final thiol for Ub_75_-thioester conjugation and DHA formation. The probe mimicked K119 ubiquitinated H2A. Nucleosome particles were reconstituted avoiding reducing conditions for DHA stability, and labeling by Calypso/ASK was confirmed. Although the methodology is powerful in the breadth of application as it could allow multiple cysteines in the peptide/protein the utility in complex systems may be limited by the native isopeptide bond.

The Zhuang laboratory used their warhead containing linker strategy to generate ubiquitin-PCNA probes representing K107 and K164 ubiquitinated PCNA. This strategy is elegant in its simplicity although it required mutation of the four cysteines in PCNA to ensure site selectivity. These probes displayed differences in the affinity enrichment of deubiquitinating enzymes in Yeast. The K164 probe enriched several DUBs whilst the K107 probe showed only modest enrichment.

## Probes for Ubiquitin Conjugation Machinery

Ubiquitin is added to substrate proteins by E1, E2, and E3 enzymes (Ciechanover et al., 1982; Hershko et al., 1983). Dysregulation of these enzymes is associated with certain cancers and neurodegenerative disorders (Bernassola et al., 2008; Popovic et al., 2014). There has therefore been a demand to develop probes for these enzymes analogous to those targeting DUBs.

### Ubiquitin-Adenine Probes

Monoubiquitin probes have been demonstrated to label ubiquitin conjugation machinery (Kamadurai et al., 2009; Love et al., 2009; Kim et al., 2011; Ekkebus et al., 2013; Maspero et al., 2013; Byrne et al., 2017) however they lack specificity. Tan and co-workers (Lu et al., 2010) developed E1 targeting probes by mimicking the adenylate intermediate formed in the E1 active site. Native chemical ligation of Ub_71_-thioester introduced the modified C-terminus of the protein. The modified C-terminus contained an electrophilic trap at the 74 position and a 5′-sulfonyladenosine-based modification. The probe labeled recombinant E1s and aided crystallization but was not tested in more complex systems. Additionally, the C-terminal ubiquitin sequence is altered and truncated which may affect selectivity. This probe design was used in subsequent studies to provide insight into structural changes within E1 enzymes during adenylation (Hann et al., 2019).

An and Statsyuk (2016) also took a native chemical ligation approach to target E1 enzymes. Flag-Ub_75_-thioester was coupled to cysteine-conjugated adenine moieties. The cysteine was then converted to DHA to furnish an electrophilic trap. The site of attack is three atoms away from the native position relative to ubiquitin and the lack of phosphate group mimic potentially reduces binding. Nonetheless, covalent labeling was observed with UBA1. The probes react specifically with their cognate E1 enzymes over Ubl conjugation machinery. However, some reactivity was observed with the DUB IsoT which also appeared to cleave the probe. This represents a limitation for cell lysate, but the probes provided an effective strategy to study E1 enzymes.

### Modified E2 Probes

More recently, Virdee and co-workers employed tosyl-substituted doubly activated enes (TDAEs) to sequentially functionalize thiols at a single carbon center for profiling E1 enzyme activity (Stanley et al., 2015). The single cysteine in E2 UBE2N was reacted with TDAEs to form E2-based probes for E1 activity. Labeling of E1 UBA1 was observed and enhanced by co-incubation with Ub and ATP. Endogenous UBA1 was selectively labeled in HEK293 lysate.

### Ubiquitin-Protein Probes for E3 Ligases

Elaborated TDAE probes aimed to specifically target the E3 ligase Parkin by incorporating ubiquitin into the probe (Pao et al., 2016). Ub_73_-thioester was reacted with azidoaminoethane to afford Ub-azide. Alkyne functionalized acrylate and acrylamide were used to prepare two TDAE functionalized ubiquitin monomers. A single cysteine mutant of His-UBE2L3 was reacted with the monomers to form the E2-Ub conjugate probes (Figure 1C). The triazole linker and electrophile replace residues 74-76 of ubiquitin. The electrophilic trap is one atom from the native position. The active site of Parkin was labeled by both probes. Furthermore, the probes were stable and inert to recombinant DUBs. Virdee et al. demonstrated an application in profiling primary fibroblasts from Parkinson's disease patients. Licchesi and co-workers (Byrne et al., 2017) later showed these probes react with NEDD4, UBE3C and HECTD1. Although not specific for a single E3, the synthetic approach is broadly applicable.

Shi and co-workers (Xu et al., 2019) designed a probe consisting of mutated UBE2D2, bearing a single cysteine, conjugated to Biotin-Ub_75_-NH_2_NH_2_ using native chemical ligation followed by Dha formation. The probes were tested against catalytic domains of NEDD4 and UBE3C, labeling both active sites. Probing HeLa cells saw enrichment of several E3 enzymes, with strong enrichment of NEDD4. This work demonstrates an alternative route to E2-Ub probes however the linker is three atoms longer than the native and the electrophilic trap is presented two and five atoms away from Ub and UBE2D2, respectively.

## Conclusion

**Figure 2:**
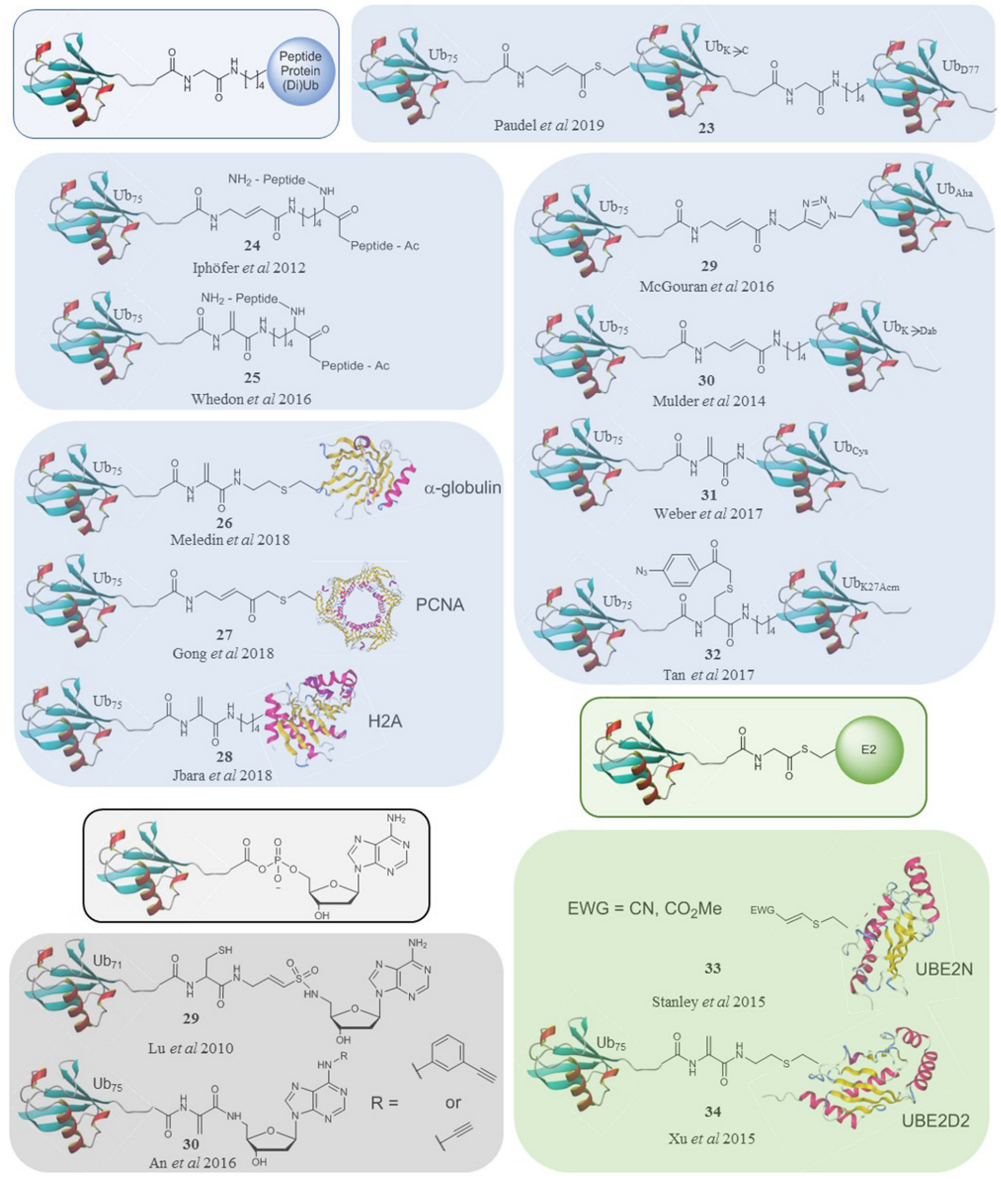
A selection of ubiquitin-based probes that specifically target subsets of DUBs (blue), E1s (black) and E3s (green) compared to the natural substrates of their targets (framed).

Several approaches have been taken to confer selectivity to activity-based probes for ubiquitin conjugation/deconjugation machinery (Figure 2). Mutation of ubiquitin has proved successful following several rounds of screening. Expansion of this method to include unnatural amino acids could prove to be powerful. Ubiquitin-peptide and diubiquitin structures, except for the OTULIN probe, did not yield probes selective for a single enzyme but they did lay much of the groundwork for generating the more complex ubiquitin-protein probes. Furthermore, di and triubiquitin probes can allow probing of ubiquitin binding pockets of DUBs beyond the S1'-S1 pocket. Several ubiquitin-protein conjugate probes now exist giving us new levels of detail. Existing and new methodology has also been applied to explore ubiquitin conjugation machinery. Selectivity was tested in recombinant and cellular systems, with probes varying in linker length, positioning of electrophilic trap and stability to DUBs as well as compatibility requirements. Many probes have proven to be excellent tools to study these complex pathways and have already provided valuable insights into the mechanistic and structural features of target enzymes (Bekes et al., 2016; Hann et al., 2019; Paudel et al., 2019) as well in the characterization of new family members (Hermanns et al., 2018) and identification of potential disease markers in patient samples (Pao et al., 2016).

Certain conjugate probes are limited due to their hydrolysable linkers and despite several well-designed solutions, many of the probes also do not perfectly mimic the linker length or trap position of the wild-type substrate. There is therefore scope for optimisation of the probe design and implementation of new chemistry for the synthesis of novel probes. Furthermore, these probes are currently limited by their lack of cell permeability. Recent work demonstrated that incorporation of cleavable cell-penetrating peptides can help deliver monoubiquitin probes into a cell (Gui et al., 2018). Application of this methodology to conjugate probes could enable the development of cell-permeable versions. Additionally, large scale biological screens combined with the latest chemoproteomic methods, similar to those carried out using monoubiquitin probes, could provide a more resolved picture of DUB activity using these more specific probes (Hewings et al., 2018; Pinto-Fernández et al., 2019). Overall, the expansion and combination of methods reviewed herein could open further possibilities, ultimately affording a panel of probes capable of targeting specific subsets or even individual enzymes. This could provide a more comprehensive view of DUB and ubiquitin conjugating enzyme activity in cells.

## Author Contributions

JM and NT drafted and edited the manuscript.

### Conflict of Interest

The authors declare that the research was conducted in the absence of any commercial or financial relationships that could be construed as a potential conflict of interest.
